# The impact of the physical activity intervention on sleep in children and adolescents with neurodevelopmental disorders: a systematic review and meta-analysis

**DOI:** 10.3389/fneur.2024.1438786

**Published:** 2024-08-13

**Authors:** Tong Wang, Weicheng Li, Jiaxin Deng, Qiubo Zhang, Yongfeng Liu, Haoyu Zheng

**Affiliations:** School of Sports Training, Chengdu Sport University, Chengdu, Sichuan, China

**Keywords:** physical activity, children and adolescents, sleep, neurodevelopmental disorders, systematic review and meta-analysis

## Abstract

**Objective:**

The purpose of this review was to synthesize the current literature on the relationship between sleep and physical activity in children and adolescents with neurodevelopmental disorders.

**Methods:**

Articles were searched in PubMed, Web of Science, EBSCO, Cochrane, and Embase until April 2024. The meta-analysis was performed using Review Manager 5.3.

**Results:**

Our results show that measuring sleep parameters by means of different measuring tools yields different results. Most studies have found no association between sleep and physical activity in children with neurodevelopmental disorders, especially when measured subjectively, such as parent reports and sleep logs. Physical activity interventions had a significant effect on sleep efficiency, wake after sleep onset, and sleep duration when measured objectively using instruments such as wrist actigraphy. Meta-analysis showed that children and adolescents with neurodevelopmental disorders who participated in mind–body activities (SMD = −3.01, 95%CI = −4.15~−1.87, *p* < 0.001, I^2^ = 99%) showed significant improvements in sleep, which were sessions lasting more than 12 weeks (SMD = −1.01, *p* < 0.01, I^2^ = 97%), performed at least 3 times per week (SMD = −0.81, 95%CI = −1.53~−0.10, *p* = 0.03, I^2^ = 95%), and lasted for more than 60 min per session (SMD = −1.55, 95%CI = −2.67~−0.43, *p* = 0.007, I^2^ = 97%). However, the results of these subgroup analyses must be interpreted with caution because of the small number of studies included.

**Conclusion:**

Our results show that measuring sleep parameters by means of different measuring tools yields different results. There was difficulty in interpreting many of the studies included in this meta-analysis, in view of the non-standardization of protocol, especially the ability range of the cohort, duration of the study, recommended exercises, whether the caregivers or researchers supervised the exercise regime/activity, and the practicality of continuing the exercise long-term by caregivers.

**Systematic review registration:**

Identifier, CRD42024541300.

## 1 Introduction

Neurodevelopmental disorders constitute a diverse array of conditions that are hallmarked by significant impairments in personal autonomy, societal engagement, and academic or vocational performance, often surfacing in the early stages of life (1). These disorders are not limited to, but encompass a range of afflictions such as autism spectrum disorder (ASD), attention-deficit/hyperactivity disorder (ADHD), intellectual disability (ID), and particular learning difficulties. The prevalence of these neurodevelopmental conditions varies across geographical regions and research studies, yet a noteworthy trend has been the escalation in occurrence rates within the past two decades. This upward trajectory underscores the importance of heightened awareness, earlier diagnosis, and the pursuit of innovative treatment modalities to address this pressing global healthcare challenge (2).

Healthy sleep plays a key role in cardiovascular, metabolic, cognitive, and maintaining good physical and mental health and is considered an important behavior for improving public health (3–5). Human sleep (and that of most mammals and birds) is composed of two distinct states: non-rapid eye movement (NREM) and rapid eye movement (REM), each with unique characteristics and each actively regulated by distinct neural centers. A typical night involves 4 to 6 repeated cycles of NREM and REM, each lasting approximately 90 to 110 min (6). REM sleep and NREM sleep are important for optimal brain development but differ in their functional contributions (7). REM sleep seems to provide the stimulation needed for preliminary development and the survival of sensorimotor neuronal networks. It does this by driving the generation of endogenous, intense, and generalized neural activity across sensorimotor systems. NREM sleep is hypothesized to contribute to brain development by optimizing neuronal networks via mechanisms of synaptic downscaling and pruning (8). Both REM and NREM stages of sleep contribute to the consolidation of nascent material (9–12). NREM 3 (N3, slow wave) sleep orchestrates hippocampal-neocortical dialog and information transfer (13, 14). Recent studies have revealed that REM sleep and REM sleep may work together to promote learning (10, 15, 16). When delving into the realm of sleep disturbances, one encounters a wide array of issues, chief among them being the reduction in overall sleep duration (17), the protracted delay in falling asleep known as sleep latency (18), and the distressing phenomenon of recurrent nocturnal awakenings (19). It is noteworthy that these maladies are often not isolated occurrences; rather, they are frequently the result of intricate interactions between neurobiological processes, psychological factors, and the social/environmental milieu. Particularly in children who are afflicted with neurodevelopmental disorders, these intersections of various factors have the capacity to significantly contribute to and exacerbate their sleep-related challenges (20, 21).

Physical activity has emerged as a promising intervention to address sleep problems, with a number of benefits that make it an accessible and widely available solution. For example, regular physical activity can improve sleep quality by reducing insomnia symptoms, prolonging sleep duration, and increasing overall sleep efficiency. The exertion of energy during physical activity not only contributes significantly to maintaining a harmonious balance of the body’s intrinsic sleep–wake cycle, but it also enhances the quality of sleep, making it more rejuvenating and restorative. This exertion of energy plays a pivotal role in regulating the body’s natural rhythm, ultimately fostering a more restful and revitalizing sleep experience (22). Martial arts have the capacity to significantly mitigate undesirable sleep behaviors in children with ASD, accomplishing this through the elicitation of hormonal, biological, and biochemical alterations within the body. As a valuable addition to a child’s daily routine, martial arts can serve as an efficacious intervention to enhance and refine sleep patterns for children with ASD, ultimately contributing to a more restful and regulated sleep experience (23). A plethora of research has delved profoundly into the intricate effects of physical activity on sleep patterns among children and adolescents afflicted with neurodevelopmental disorders. For instance, the eminent study conducted by Tse and his team in 2019 provides profound insights into this matter. This study encompassed a rigorous 12-week basketball training program, encompassing 40 children diagnosed with ASD. The results were compelling, revealing that those children who actively participated in the basketball training program exhibited marked improvements in sleep efficiency, coupled with a substantial reduction in the duration of wakefulness after initially falling asleep. In stark contrast, the control group, who did not undergo the training, did not experience such noteworthy benefits. This study underscores the profound potential of physical activity, particularly basketball training, in enhancing sleep patterns among children with ASD (24). Furthermore, Tse et al. (25) conducted a comprehensive study that highlighted the profound effects of a 12-week morning jogging intervention on the sleep patterns of children with ASD. This intervention involved two 30-min sessions each week, and the results were nothing short of remarkable. The study revealed a significant improvement in sleep efficiency and an extension in sleep duration for the participating children. This finding underscores the positive and profound correlation between regular physical activity and the enhancement of sleep quality among this vulnerable group of children. By incorporating physical exercise into their daily routines, children with ASD can significantly improve their sleep patterns, ultimately contributing to their overall health and well-being (26).

To our knowledge, no reviews have examined the effects of physical activity interventions on sleep in children and adolescents with neurodevelopmental disorders. Therefore, this review aimed to synthesize published research on the effects of physical activity interventions on sleep in children and adolescents with neurodevelopmental disorders.

## 2 Methods

Following the guidelines outlined in the Preferred Reporting Items for Systematic Reviews and Meta-Analysis (PRISMA) (27) and the Cochrane handbook for systematic reviews and meta-analysis (28), this review was conducted. Moreover, the protocol for this review was duly registered on PROSPERO under the registration number CRD42024541300.

### 2.1 Search strategy

Five databases—Web of Science, EBSCO, PubMed, Cochrane, and Embase—were searched to identify relevant literature. The search date was April 2024. These databases were chosen based on their related content areas in children and adolescents with neurodevelopmental disorders and physical activity. A summary of the search terms is shown in Table 1.

**Table 1:** Summary of search terms.

Category		Included search terms
Physical activity		(“physical activity,” “physical exercise,” “sports activities, “physical education,” motor, “sport movement,” sport, “athletic sports,” “aerobic activity,” “aerobic training,” “resistance exercise,” “muscle-strengthening exercise,” “strength training,” “fitness game,” “Sports game”)
	AND	
Neurodevelopmental disorder		(“neurodevelopmental disorder,” “autism spectrum disorder,” autism, “asperger syndrome,” asperger, “pervasive developmental disorder not otherwise specified,” “attention-deficit/hyperactivity disorder,” ASD, ADD, ADHD, “intellectual disability,” “down syndrome,” “motor disorder,” “specific learning disorder,” “developmental coordination disorder,” “learning disability,” “communication disorder,” “developmental disorder”)
	AND	
Sleep		(sleep, “sleep health,” “sleep duration,” “sleep quality,” polysomnography)
	AND	
Children and adolescents		(children, adolescents, child, teenager)

### 2.2 Eligibility criteria

The relevant studies’ inclusion criteria were established following the PICOS framework. Participants (P) were children and adolescents aged 5–18 years who had been diagnosed with a neurodevelopmental disorder (i.e., ASD, ADHD, ID, and learning disorders). Intervention (I): the experimental group received various forms of physical activity interventions. Comparison (C) groups encompassed no treatment or no exercise group (NT), waitlist (WL), usual care and daily activities (UC), and attention/activity placebo conditions (AP). Outcome (O) measurements focused primarily on sleep problems in neurodevelopmental disorders. The sleep measurement tools were one of actigraphy, parent-reported questionnaires, and sleep logs. Lastly, Study Design (S) adhered to the randomized controlled trial methodology or non-randomized comparison studies (NRS) and reported sufficient statistical details (e.g., mean, SD, number of participants) were included in the meta-analysis. During the meta-analysis, we excluded studies that lacked a comparison group or failed to report comparative outcomes between groups (e.g., single-case studies or pre-experimental designs).

The following criteria were applied to exclude relevant studies: literature not in English, including unpublished materials, theses, and reviews; studies involving adults or animals; literature lacking valid data extraction; duplicate publications; and full texts that were inaccessible.

### 2.3 Data extraction

Following the intervention, the focus was assessing neurodevelopmental disorders. Utilizing Review Manager 5.3 (28), data were inputted for both intervention and control groups, encompassing mean values, standard deviations, and participant counts. To accommodate the anticipated heterogeneity between trials, attributed to the implementation of diverse physical activity interventions, meta-analysis pooling was conducted using a random-effects model. To facilitate the aggregation of data from various depression symptom scales, the effect was evaluated as the standardized mean difference (SMD), calculated using Hedges’ g, adjusted for small sample size bias, accompanied by 95% confidence intervals (CI) (29). Heterogeneity was evaluated through standard parameters of the I^2^ statistic (28). In cases where the test indicated substantial heterogeneity (I^2^ > 50%), we employed subgroup analysis or sensitivity analysis to elucidate the findings (30). Due to the inclusion of fewer than 10 studies in each analysis, a comprehensive investigation of potential publication bias was deemed unwarranted.

### 2.4 Methodological quality assessment

We assessed the methodological quality of each included study according to the Physiotherapy Evidence Database scale (31), a reliable and valid tool for assessing the methodological quality of RCT and NRS studies. Previously, PEDro demonstrated its validity in evaluating studies of physical activity interventions for children with neurodevelopmental disorders (32, 33). The scale includes 11 rating criteria on eligibility, randomization, allocation, and blinding (34). Scores range from 0 to 10. In addition, a previous review noted that in many physical activity intervention trials, blinding may sometimes not be possible. Therefore, blinding subjects and therapists in physical activity interventions and obtaining two scores can be challenging (35). In light of the inherent constraints associated with physical activity interventions, the scoring system was meticulously segmented into three distinct categories, in alignment with preceding evaluations. Specifically, a score of 6 or above was deemed to represent a study of superior quality, indicating rigor and comprehensiveness. A score ranging from 4 to 5 was classified as sufficiently high quality, reflecting adequate standards in the research methodology. A score of 3 or below was indicative of a study of inferior quality, suggesting areas for improvement or refinement in the research design. This categorization scheme enables us to accurately assess the quality of the interventions and provides valuable insights for future research endeavors.

## 3 Results

### 3.1 Study selection

**Figure 1:**
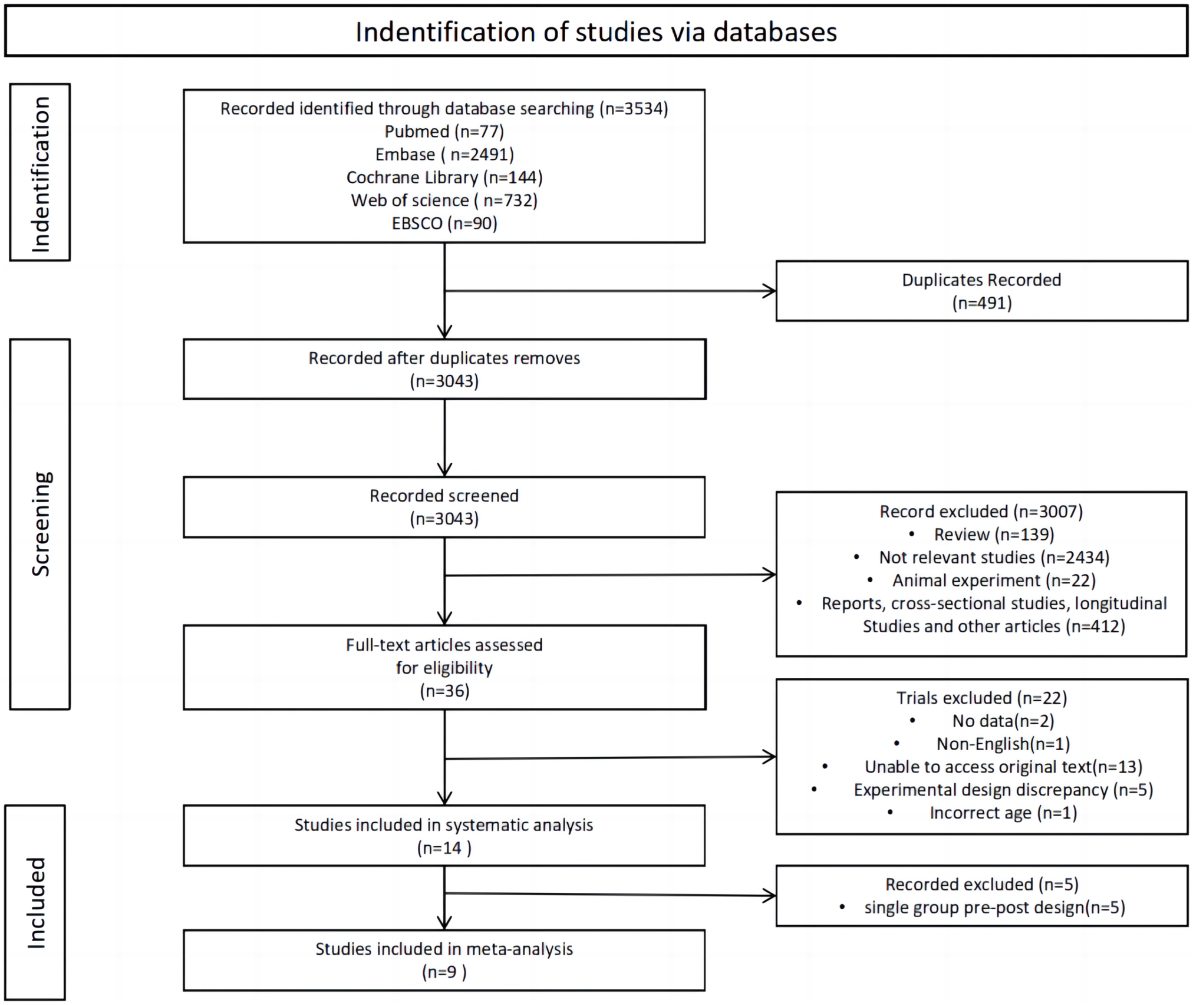
Flow chart of literature retrieval.

The review search results and research selection process are shown in Figure 1. Initially, 3,534 articles were retrieved from the databases. Following the removal of duplicates, 3,043 studies remained, while 3,007 studies did not meet the eligibility criteria during the title and abstract screening phase. Out of 36 studies, 22 were excluded after reading their full texts: (1) no data (*n* = 2); (2) non-English (*n* = 1); unable to access original text (*n* = 13); (4) experimental design discrepancy (*n* = 5); and (5) Incorrect age (*n* = 1). A systematic analysis encompassed a total of 14 articles, and after excluding single-group pre-post design, the remaining 9 were included in the meta-analysis.

### 3.2 Study characteristics

The characteristics of the included studies are shown in Table 2. The sources of selection participants included special schools (*n* = 6) (24, 26, 36–39), special institutions (*n* = 3) (22, 23, 40), special classroom (*n* = 1) (41), and pediatric center (*n* = 1) (42) (“*n*” Numbers refer to the number of papers), in addition to three studies that did not report on the sources of selection participants (43–45). The age range of children and adolescents included in the study was 2.3–18 years. The types of children and adolescents with neurodevelopmental disorders included ASD (*n* = 12) (22–24, 26, 38–45), ADHD (*n* = 1) (37), and ID (*n* = 1) (36). Diagnostic Methods include DSM-5 (*n* = 8) (24, 36, 37, 39, 40, 42, 44, 45), ICD-10 (*n* = 2) (22, 38), Parent report (*n* = 1) (41), ADOS-2 (*n* = 1) (26), GARS-2 (*n* = 1) (23). Intervention designs fall into three categories: water-based aerobics (e.g., swimming) (40, 41, 44, 45), mind–body activities (e.g., Yoga and Karate) (23, 38, 43) and land-based aerobics (e.g., basketball, cycling and jogging) (22, 24, 26, 36, 37, 39, 42). Intervention duration ranged from 3 to 48 weeks, and intervention frequency ranged from 1 to 5 times per week, with each session lasting from 30 to 120 min. As for the timing of activity, only five studies reported information on this, divided mainly into morning (24, 26, 38) and afternoon (40, 43) periods.

**Table 2:** Descriptive characteristics of included studies.

Included studies	Research design	Sample size (IG/CG)	Age range	Diagnosis/diagnostic method	Source of participants	Timing	Intervention design	Intensity	Duration/Time/Frequency	Outcome Measures	Activity the control group
Adib Saber et al., 2021^*^	RCT	20(10/10)	8–14	ASD; GARS-2	Support institutions	-	Karate (mind–body activities)	-	10 week; 60-min/session; 2 times/week	CSHQ	UC
Ansari et al., 2021^*^	RCT	40(20/20)	6–14	ASD; DSM-5	Support institutions	Afternoon	Swimming (water-based aerobics)	-	10 week; 60-min/session; 2 times/week	CSHQ	NT
Brand et al., 2015	PPT	10	7–13	ASD; ICD-10	Support institutions	-	Bicycle and motor skills (land-based aerobic exercise)	-	3 week; 60-min/session; 3 times/week	Sleep-EEG	-
										PSQI	
Garcia et al., 2024	PPT	18	Mean age: 13.17 ± 3.76	ASD; −	-	Afternoon	Family judo program (mind–body activities)	-	14 week; 45-min/session; 1 times/week	Actigraph GT9X accelerometer	-
Gudek Seferoglu, 2022^*^	RCT	126(62/64)	8–18	ID; DSM-V	Special school	-	Volleyball, basketball, et al. (land-based aerobics)	Mod	30 week; 120-min/session; 3 times/week	CSHQ	NT
Lawson et al., 2017	PPT	10	5–12.3	ASD; DSM-5	-	-	Swimming (water-based aerobics)	-	8 week; 30-min/session; 1 times/week	CSHQ	-
Lawson et al., 2022	PPT	3	4–17	ASD; DSM-5	-	-	Swimming (water-based aerobics)	-	8 week; 30-min/session; 1 times/week	Beddit Sleep Monitor	-
										CSHQ	
										Sleep log	
Liu et al., 2023^*^	RCT	33(17/16)	8–12	ADHD; DSM-5	Special school	-	Jogging (land-based aerobics)	Mod	12 week; 45-min/session; 1 times/week	Actigraphy	NT
										Sleep log	
NaraSiNgharao et al., 2017^*^	NRS	64(32/32)	5–16	ASD; ICD-10	Special school	Morning	Yoga (mind–body activities)	-	90 sessions; 75-min/session; 5 times/week	Sleep questionnaire for parents	-
Oriel et al., 2016	PPT	8	6–11	ASD; Parent report	Support classrooms	-	Aquatic (water-based aerobics)	Mod	4 week; 60-min/session; 2 times/week	CSHQ	-
										Sleep-related questions	
Toscano et al., 2022^*^	NRS	229 (127/62/40)	2.3–17.3	ASD; DSM-5	Pediatric center	-	Coordination and strength exercise (land-based aerobics)	Mod	48 week; 40-min/session; 2 times/week	Autistic traits assessment scale	-
Tse et al., 2019^*^	RCT	40 (19/21)	8–12	ASD; DSM-5	Special school	Morning	Basketball (land-based aerobics)	Mod	12 week; 45-min/session; 2 times/week	Actigraphy	NT
										Sleep log	
Tse et al., 2022^*^	RCT	55 (23/32)	8–12	ASD; ADOS-2	Special school	Morning	Jogging (land-based aerobics)	Mod	12 week; 30-min/session; 2 times/week	Actigraphy	NT
										Sleep log	
Tse et al., 2023^*^	RCT	36 (18/18)	8–12	ASD; DSM-5	Special school	-	Cycling (land-based aerobics)	-	2 week; 60-min/session; 5 times/week	GT3X accelerometer	AP
										Actigraphy	
										Sleep log	

AP, attention/activity placebo conditions; CG, control group; CSHQ, Children’ s Sleep Habits Questionnaire; IG, intervention group; Mod, Moderate; NRS, non-randomized comparison study; NT, not exercise group; PPT, pre-post-test; PSQI, Pittsburgh Sleep Quality Index; RCT, Randomized Control Trial; UC, usual care and daily activities; −, no report; ^*^Studies included in the meta-analysis.

### 3.3 Study designs

A total of five studies used a single-group before-and-after trial design (22, 41, 43–45); nine studies used an RCT or quasi-experimental design, seven of which used an RCT design (23, 24, 26, 36, 37, 39, 40) and two of which used an NRS design (38, 42). The activity profile of the control group in the 7 RCT studies was no exercise program (*n* = 5) (24, 26, 36, 37, 40), regular treatment and daily physical activity (*n* = 1) (23), and placebo group (*n* = 1) (39). However, monitoring of the control group was not reported in any of the seven RCT studies.

### 3.4 Types of sleep assessments

Eight research studies (23, 36, 38, 40–42, 44, 45) relied on questionnaires or surveys reported by parents to evaluate the impact of physical activity interventions on sleep patterns. These assessments provided valuable insights into the subjective experiences of sleep quality and quantity. Meanwhile, seven studies (22, 24, 26, 37, 39, 43, 44) opted for objective sleep monitoring methods, such as actigraphy and sleep EEG, to scientifically measure sleep patterns and duration. In addition, four studies (24, 26, 37, 39) relied on sleep logs to assess the effects of physical activity interventions on sleep. Notably, six studies (23, 36, 40, 41, 44, 45) specifically selected the Children’s Sleep Habits Questionnaire (CSHQ), a parent-reported survey, as their preferred tool for assessing sleep habits in children and adolescents diagnosed with neurodevelopmental disorders. This choice underscores the questionnaire’s reliability and validity in this specific population.

### 3.5 Quality assessment

Table 3 presents a refined evaluation of the methodological rigor and quality of the studies incorporated in our analysis. Notably, all studies exhibited adherence to at least three key criteria, thereby demonstrating a fundamental level of scientific rigor. Among them, eight studies employed an investigative trial design, reflecting a particularly high study quality. Overall, the studies included in the meta-analysis exhibited satisfactory quality, with a mean score of 5.67, indicative of a robust methodological foundation. Each study clearly defined its recruitment criteria, thus ensuring the selection of participants well-suited to the research objectives. Moreover, the retention rates during the intervention period were commendably high, minimizing attrition and preserving the integrity of the data. However, we observed that only a limited number of studies successfully implemented blinding procedures for participants through randomized sequence generation, a crucial aspect in controlling for potential biases. Additionally, only three studies adequately blinded their outcome assessors, a measure that further enhances the objectivity and reliability of the research findings.

**Table 3:** Methodological quality assessment for included studies.

Included studies	Eligibility criteria	Random allocation	Allocation concealment	Similar at baseline	Subject blinded	Therapist blinded	Assessor blinded	Dropout rate	Intention-to-treat analysis	Between-group comparison	Points measures	Total score	Overall study quality
Adib Saber et al., 2021^*^	1	1	1	1	0	0	0	0	0	1	1	5	Adequate
Ansari et al., 2021^*^	1	1	0	1	0	0	0	1	0	1	1	5	Adequate
Brand et al., 2015	1	0	0	1	0	0	0	1	0	0	1	3	Poor
Garcia et al., 2024	1	0	0	1	0	0	0	1	0	0	1	3	Poor
Gudek Seferoglu et al., 2022^*^	1	1	1	1	0	0	0	1	0	1	1	6	High
Lawson et al., 2017	1	0	0	1	0	0	0	1	0	0	1	3	Poor
Lawson et al., 2022	1	0	0	1	0	0	0	1	0	0	1	3	Poor
Liu et al., 2023^*^	1	1	1	1	0	0	0	1	0	1	1	6	High
NaraSiNgharao et al., 2017^*^	1	0	0	1	0	0	0	1	0	1	1	4	Adequate
Oriel et al., 2016	1	0	0	1	0	0	0	1	0	0	1	3	Poor
Toscano et al., 2022^*^	1	0	0	1	0	0	0	1	0	1	1	4	Adequate
Tse et al., 2019^*^	1	1	1	1	0	0	1	1	0	1	1	7	High
Tse et al., 2022^*^	1	1	1	1	0	0	1	1	0	1	1	7	High
Tse et al., 2023^*^	1	1	1	1	0	0	1	1	0	1	1	7	High

^*^Yes = 1; No = 0. ≤3 are considered “poor,” 4–5 are considered “adequate,” ≥6 are high. ^*^Study included in the meta-analysis.

### 3.6 Meta-analysis

Among the total of 14 studies evaluated, nine—comprised of seven RCTs and two NRSs—emerged as eligible for a comprehensive meta-analysis.

**Figure 2:**
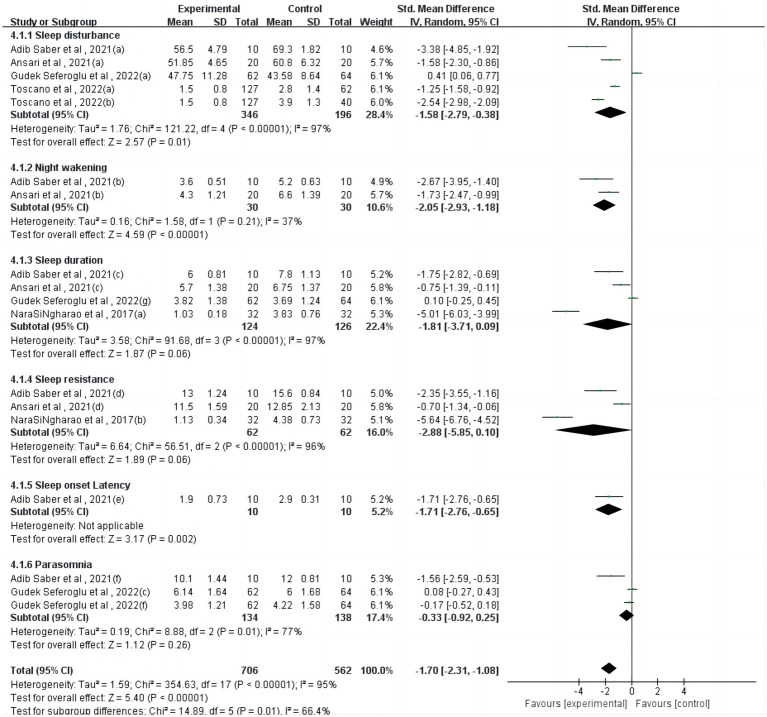
Forest plot for meta-analysis of parent-reported sleep questionnaire.

Regarding the parent-reported sleep questionnaire (Figure 2), one study (23) reported a significant but heterogeneous effect of physical activity on sleep onset latency (*p* = 0.002). However, physical activity interventions showed non-significant effects on sleep duration (*p* = 0.06), sleep resistance (*p* = 0.06), and parasomnia (*p* = 0.26).

**Figure 3:**
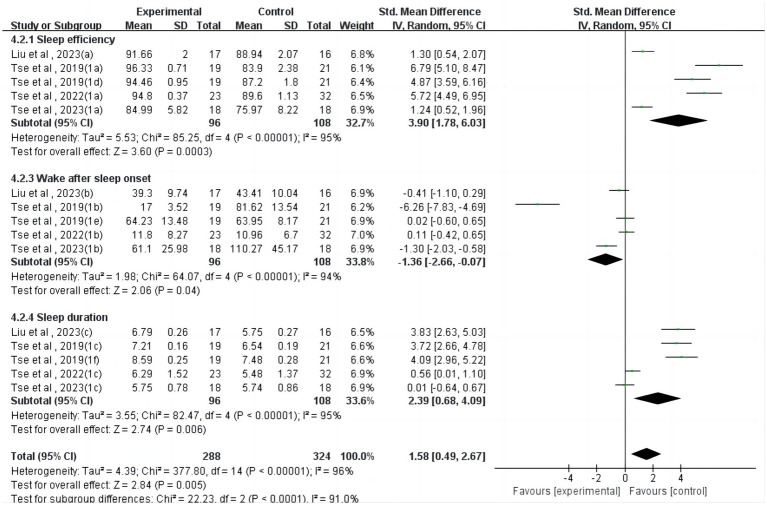
Forest plot for meta-analysis of actigraphy-measured sleep parameters.

Regarding actigraphy (Figure 3), four studies (24, 26, 37, 39) reported a significant but heterogeneous effect of physical activity on sleep efficiency (SMD = 3.90, 95%CI = 1.78 ~ 6.03, *p* < 0.001, I^2^ = 95%). Four studies (24, 26, 37, 39) reported a significant but heterogeneous effect of physical activity on wake after sleep onset (SMD = −1.36, 95%CI = −2.66~−0.07, *p* < 0.05, I^2^ = 94%). Four studies (24, 26, 37, 39) reported a significant but heterogeneous effect of physical activity on sleep duration (SMD = 2.39, 95%CI = 0.68 ~ 4.09, *p* < 0.01, I^2^ = 95%).

**Figure 4:**
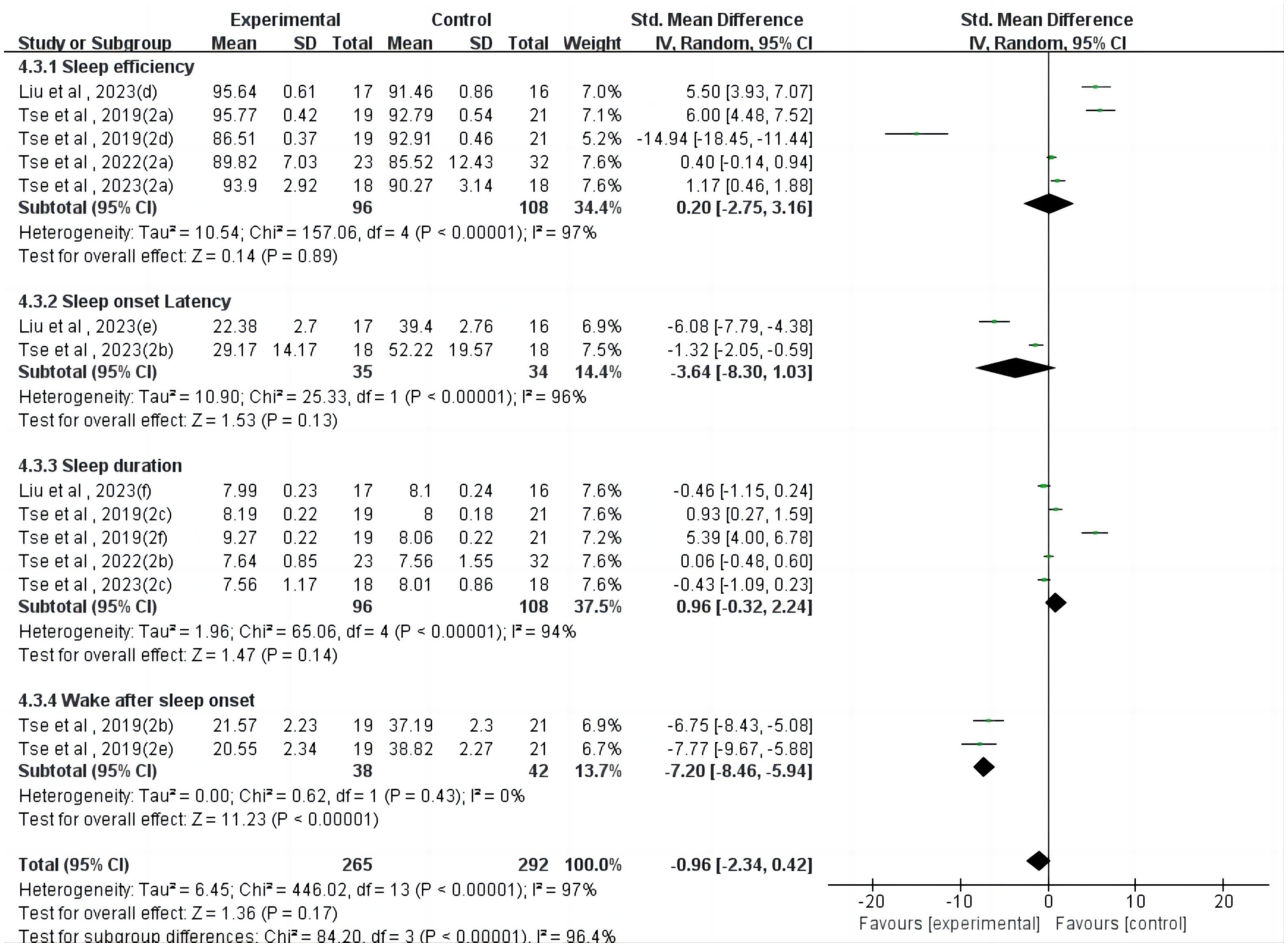
Forest plot for meta-analysis of sleep log reports.

Regarding sleep logs (Figure 4), one study (24) reported a significant effect of physical activity on wake after sleep onset (SMD = −7.20, 95%CI = −8.46~−5.94, *p* < 0.001, I^2^ = 0%). However, physical activity interventions showed non-significant effects on sleep efficiency (*p* = 0.89), sleep onset latency (*p* = 0.13), and sleep duration (*p* = 0.14).

### 3.7 Sensitivity analysis

Conducting sensitivity analysis on the encompassed studies revealed that the manipulation of individual references did not yield substantial alterations to the outcome metrics. This suggests that the findings derived from this meta-analysis exhibit stability and reliability.

### 3.8 Subgroup analysis

The subgroup targets were activity intervention design (water-based aerobics vs. mind–body activities vs. land-based aerobics), activity intervention duration (≤12 weeks vs. >12 weeks), activity intervention time (<45 min vs. 45–60 min vs. >60 min), activity intervention frequency (<3 times per week vs. ≥3 times per week), activity timing (morning vs. afternoon).

#### 3.8.1 Physical activity intervention design

One study (40) provided data for water-based aerobics, two studies (23, 38) provided data for mind–body activities, and six studies (24, 26, 36, 37, 39, 42) provided data for land-based aerobics. Subgroup analyses showed that mind–body activities (SMD = −3.01, *p* < 0.001, I^2^ = 88%) significantly improved sleep in children and adolescents.

#### 3.8.2 Physical activity intervention duration

Six studies (23, 24, 26, 37, 39, 40) provided data for intervention duration of less than or equal to 12 weeks, and three studies (36, 38, 42) provided data for more than 12 weeks. The results showed that when the duration was more than 12 weeks (SMD = −1.64, *p* < 0.001, I^2^ = 98%) the activity intervention had a significant impact on children and adolescents’ sleep. When the duration was less than or equal to 12 weeks (*p* = 0.67), the physical activity intervention had no significant effect on sleep in children and adolescents.

#### 3.8.3 Physical activity intervention time

Two studies (26, 42) provided data for intervention time of less than 45 min, five studies (23, 24, 37, 39, 40) provided data for intervention time of 45–60 min, and two studies (36, 38) provided data for intervention time of over 60 min. The results showed that physical activity interventions had a significant effect on sleep in children and adolescents when the duration of the intervention was more than 60 min of long time (SMD = −1.55, *p* <0.01, I^2^ = 97%). When the duration of the physical activity intervention was short (*p* = 0.57) and medium time (*p* = 0.34), the effect on sleep was not significant. Therefore, the physical activity of adolescents can be controlled over 60 min.

#### 3.8.4 Physical activity intervention frequency

Six studies (23, 24, 26, 37, 40, 42) provided data at a frequency of less than 3 times per week, and three studies (36, 38, 39) provided data at a frequency of at least 3 times per week. The results showed that adolescents with at least 3 times per week of physical activity intervention (SMD = −0.81, *p* <0.05, I^2^ = 95%) could better improve sleep, and those with less than 3 times a week of physical activity intervention (*p* = 0.48) could also improve sleep, but the effect was not as significant as that with more than 3 times a week of physical intervention. Therefore, children and adolescents should try to control physical activity at least 3 times a week.

#### 3.8.5 Activity timing

Three studies (24, 26, 38) recorded morning timing of activity, and one study (40) exercise was scheduled for afternoon. There was a significant effect on improving sleep in children and adolescents with neurodevelopmental disorders when the activity was scheduled in the afternoon (*p* < 0.001) and no significant effect on improving sleep in children and adolescents with neurodevelopmental disorders when it was scheduled in the morning (*p* = 0.77). However, due to the variation in study protocols and small sample size, the findings should be interpreted with caution.

## 4 Discussion

This study aimed to explore how physical activity affects sleep in children and adolescents with neurodevelopmental disorders, and of the initial 3,534 articles, 14 studies were deemed eligible for inclusion in the systematic review, and 9 were included in the subsequent meta-analysis.

### 4.1 Measurement of sleep and physical activity

Three outcome indicators, such as parent-reported sleep questionnaire, actigraphy-measured sleep parameters, and sleep log.

From the parent-reported sleep questionnaire, physical activity had a significant effect on sleep onset latency (*p* = 0.002), but physical intervention had a non-significant effect on sleep duration (*p* = 0.06), sleep resistance (*p* = 0.06), and parasomnia (*p* = 0.26), which were not significant. For example, in one study, researchers compared the quality of sleep and quality of life of children who did or did not participate in regular physical activity (36). There were no significant differences in sleep patterns or sleep habits between the two groups. Because self-report methods inherently have a tendency to under- or overestimate sleep duration (e.g., it is difficult to predict when a person has fallen asleep), more and more people are adopting device-based measures of sleep to provide more accurate estimates (46).

In terms of actigraphy-measured sleep parameters, physical activity had a significant effect on sleep efficiency (*p* < 0.01), wake after sleep onset (*p* < 0.05), and sleep duration (*p* < 0.01). In a previous comprehensive analysis, it was observed that there was no significant disparity in the sleep patterns of children diagnosed with ASD and those without the condition, specifically when assessed using actigraphy for measures such as sleep efficiency, overall sleep duration, and Wake After Sleep Onset. This finding indicates that in terms of these specific sleep metrics, children with ASD do not exhibit distinct differences compared to their peers without the disorder (47); in a more nuanced assessment, two separate meta-analyses have converged to affirm that children diagnosed with ASD exhibit distinct sleep characteristics compared to their peers without the condition. Specifically, these studies have demonstrated that children with ASD tend to experience longer sleep latency, shorter overall sleep duration, and decreased sleep efficiency. This discrepancy in sleep patterns highlights the unique challenges faced by children with ASD in achieving and maintaining restful sleep (48, 49). Although our review confirmed a significant effect of physical activity intervention on sleep efficiency (*p* < 0.01), wake after sleep onset (*p* < 0.05), and sleep duration (*p* < 0.01) measured by actigraphy in children and adolescents with neurodevelopmental disorders, this was mainly due to only two studies having small sample sizes, which affected the validity and reliability of the results.

In addition, from the sleep log, the physical activity intervention had a significant effect on wake after sleep onset (*p* < 0.01), but the physical activity intervention did not have a significant effect on sleep efficiency (*p* = 0.89), sleep onset latency (*p* = 0.13), and sleep duration (*p* = 0.14) had no significant effects. In sleep log assessment, participants recall specific sleep patterns (e.g., bedtime, sleep onset time, sleep end time, wake time, and sleep duration) with the help of their parents and record them in a sleep log throughout the assessment period. However, such an assessment is more subjective and may be prone to errors due to recall bias or inconsistent recording using this method, which may affect the accuracy and reliability of the data collected.

### 4.2 Meta-analytic findings

Further subgroup analyses showed that children and adolescents with neurodevelopmental disorders who participated in mind–body activities showed significant improvements in sleep, which were sessions lasting more than 12 weeks, performed at least 3 times per week and more, and lasted for more than 60 min per session. However, the results of these subgroup analyses must be interpreted with caution because of the small number of studies included.

Firstly, we categorized the included studies into three subgroups based on intervention type: water-based aerobics, mind–body activities, and land-based aerobics. Further subgroup analyses showed that children and adolescents who participated in mind–body activities (e.g., karate, yoga, etc.) showed significant improvements in sleep. The value of mind–body activities such as Tai-chi and yoga in improving sleep in children with ADHD (50) and older adults (51) has also been shown in other studies. Indeed, it is possible that regular PA, regardless of the type of PA, has the potential to improve sleep quality (52). In this study, water-based intervention also significantly enhanced sleep (*p* < 0.001). However, as only one study on water-based aerobics was included in the subgroup analyses, the results may not accurately reflect the entire subgroup population and should therefore be interpreted with caution.

Secondly, we categorized the included studies into two subgroups based on intervention duration: less than or equal to 12 weeks and more than 12 weeks. Subgroup analyses showed that more than 12 weeks of activity significantly improved sleep in children and adolescents with neurodevelopmental disorders. The value of longer-duration intervention is best shown in one study included in our meta-analyses (38) which involved a yoga program maintained for 3 months. Moreover, it profoundly enhanced sleep quality and duration. Compared to before the intervention, children exhibited remarkable improvements, such as sleeping longer without disturbances, adhering to a regular sleep schedule, and maintaining wakefulness throughout the day. These positive changes not only benefited the children but also enabled parents and other family members to enjoy a restful night’s sleep (38). In addition, another study showed that children and adolescents who received a 48-week exercise intervention showed substantial improvements in sleep disturbances. In contrast, participants in both control groups showed a trend toward increased sleep disturbances (42). Thus, this observation confirms the recommendation that clinical professionals should implement physical activity interventions for children with ASD, especially for those with sleep disorders and low medication adherence (22, 53).

Thirdly, based on the time of the intervention, we categorized the included studies into three subgroups: short (<45 min), medium (45–60 min), and long (>60 min). Studies with an intervention duration of less than 60 min and studies with an intervention duration greater than or equal to 60 min. Our subgroup analyses showed that more than 60 min per physical activity intervention significantly improved sleep in children and adolescents with neurodevelopmental disorders, whereas physical activity interventions lasting less than 60 min had no significant effect on improving sleep in children and adolescents. Certainly, the esteemed 2020 World Health Organization Guidelines on Physical Activity and Sedentary Behaviors have issued an unequivocal recommendation for children and adolescents, inclusive of those with disabilities like ASD, to engage in a minimum of 60 min of physical activity per day in order to reap significant health benefits. This recommendation underscores the vital importance of physical activity for the holistic wellbeing of individuals, regardless of their ability status (54). However, children and adolescents with ASD often fail to meet WHO recommended guidelines for MVPA (55). Furthermore, the significance of physical activity and sleep has been increasingly acknowledged as integral components of the 24-h exercise continuum, thus emphasizing the importance of a balanced lifestyle. Consequently, it is recommended that children aged 5 to 17 years should aim for a sufficient sleep duration ranging from 8 to 11 h, coupled with a minimum of 60 min of moderate-to-vigorous physical activity (MVPA) per day. This holistic approach ensures that children are able to maintain optimal physical and mental health (56). Finally, we categorized the included studies into two subgroups according to intervention duration: a subgroup of less than three times per week and a subgroup of three or more times per week. Subgroup analyses indicated that physical activity three or more times per week was more effective than physical activity less than three times per week. One study showed marked enhancements in sleep efficiency, onset latency, and duration following weekly cycling. Participants achieved optimal sleep utilization, faster fall asleep times, and longer, more restorative sleep (39).

As for the timing of activity, our meta-analysis showed significance (*p* < 0.001) when exercise was scheduled in the afternoon, but with only one study, it may not be representative. When scheduled in the morning, it was not significant (*p* = 0.77). However, previous studies have shown that the timing of exercise significantly impacts sleep, with varying effects depending on whether exercise is performed in the morning, afternoon, or evening. Morning workouts can align circadian rhythms, enhance sleep quality, and increase sleep duration, while also boosting daytime alertness and mood, thus supporting better sleep (57, 58). Afternoon exercise reduces sleep onset latency and increases total sleep time, benefiting those with an afternoon circadian preference (59). Evening exercise’s effects are more variable and depend on intensity and individual differences, with high-intensity evening exercise potentially delaying sleep onset and reducing sleep efficiency for some (60). Reviews indicate that while exercise generally promotes better sleep, the timing and individual variability are crucial factors (61). Therefore, further evidence is required to elucidate the relationship between exercise timing and sleep, particularly in children and adolescents with sleep disorders like autism.

### 4.3 Relationship between sleep and physical activity

The dynamics of sleep regulation are intricate and interactive. Numerous studies have shown that serotonin, an important neurotransmitter, plays a key role in the formation of sleep patterns in patients with ASD (62). Notably, reduced serotonin levels in the brains of these patients have been associated with a variety of sleep disorders, including difficulty falling asleep and frequent nocturnal awakenings (20). In addition, children with concurrent neurodevelopmental disorders often add another layer of complexity to their sleep patterns. This is especially troubling in children with autism, whose heightened sensitivity to external noise often disrupts their sleep. This is further exacerbated by short sleep duration and inconsistent sleep patterns that result in irregular sleep onset and wake cycles. This irregularity can adversely affect their overall health, cognitive abilities, and emotional stability. The potential for exercise to enhance sleep quality among children and adolescents with neurodevelopmental disabilities is vast and multifaceted.

Firstly, for children and adolescents with neurodevelopmental disorders, the current study found that physical activity improves sleep in children with ADHD may be due to the fact that the main drugs used to treat ADHD and physical activity both act on the catecholamine pathway, and that exercise causes an increase in norepinephrine (63), a catecholamine hormone that can regulate arousal and thus improve sleep in children with ADHD (64); exercise also induces an increase in 5-hydroxytryptamine (65), which regulates aggressive and hyperactive behaviors in children with ADHD, thereby assisting sleep (66). Currently, the mechanism of sleep improvement by exercise is limited to a single neurobiological mechanism, and the existence of higher-order interactive synergistic effects needs to be further investigated. Secondly, it is crucial to recognize that those with disabilities who struggle with sleep issues have a particularly high stake in the success of sleep health promotion efforts. Their condition often predisposes them to sleep difficulties, and thus, they stand to gain significantly more from interventions aimed at improving sleep quality. This is because, for those starting from a lower baseline, the scope for improvement is much broader. In contrast, those without disabilities who already enjoy good sleep may find it difficult to achieve further gains (67).

Secondly, the integration of physical activity into the daily routine of these children and adolescents is essential for promoting their holistic health. Physical activity not only builds strong bodies and improves physical fitness but also brings numerous psychological and emotional benefits. Among these, its ability to regulate circadian rhythms in individuals with neurodevelopmental disorders, particularly autism, is particularly noteworthy. Circadian rhythms, which govern our sleep–wake cycle, body temperature, hormone secretion, and other physiological functions, are intricately linked to melatonin levels. Melatonin, a hormone produced by the pineal gland in the brain, plays a crucial role in regulating sleep. Its production and release follow a circadian rhythm, peaking in the evening and facilitating sleep onset (68). Research has revealed that individuals with neurodevelopmental disorders, including autism, often have abnormal melatonin levels. Specifically, over 65% of them have melatonin levels significantly below the norm (69, 70). This deficit in melatonin production can contribute to sleep difficulties, such as delayed sleep onset and early morning awakenings. Children and adolescents with neurodevelopmental disorders tend to have a lesser increase in melatonin levels during the night compared to their unaffected peers, further exacerbating their sleep issues (71). Fortunately, physical activity interventions have proven to be effective in modulating melatonin levels in this specific group. By increasing physical activity levels, these children and adolescents can experience a boost in melatonin production (72). For example, a morning running program has been found to improve sleep efficiency among children with autism. Not only did they fall asleep faster and sleep more soundly, but they also experienced a significant increase in melatonin levels. This increase in melatonin levels, in turn, facilitated their sleep onset and consolidation, leading to an overall improvement in sleep quality (26).

The mechanism behind this improvement lies in the influence of physical activity on the circadian rhythm of melatonin production. By engaging in regular physical activity, these children and adolescents can synchronize their circadian rhythms and melatonin production, thus enhancing their sleep quality. This positive feedback loop of physical activity promoting better sleep and better sleep-enhancing physical and cognitive performance is particularly important for individuals with neurodevelopmental disabilities. In conclusion, the potential for exercise to enhance sleep quality among children and adolescents with neurodevelopmental disabilities is immense. By promoting physical activity and synchronizing circadian rhythms, we can help these children and adolescents overcome their sleep difficulties and enjoy a better quality of life. It is crucial for parents, educators, and healthcare professionals to recognize the importance of physical activity in promoting sleep health among this vulnerable population and to incorporate it into their treatment plans accordingly.

### 4.4 Limitations of the review

This review has several limitations. Firstly, the scarcity of studies and samples encompassed within this review may potentially yield inconclusive findings regarding the efficacy of physical activity interventions. Secondly, in some studies, researchers solely relied on diverse parent-report questionnaires as their primary measurement instruments for assessing sleep patterns in children and adolescents with neurodevelopmental disorders. However, this approach may be prone to inaccuracies stemming from recall biases or inconsistencies in recording, potentially compromising the accuracy and reliability of the gathered data. Thirdly, due to the limited inclusion of fewer than 10 studies in this review, certain subgroup analyses and potential moderators, such as the severity of neurodevelopmental disability among children and adolescents or the impact of the intensity of physical activity interventions, may not have been examined and identified. Fourthly, the children and adolescents with neurodevelopmental disorders included were predominantly ASD, with one study in ADHD children and adolescents, and future relevant research should increase the number of studies related to the relationship between other neurodevelopmental disorders and sleep in children and adolescents. Fifthly, future research protocols should include the time of day for exercise (e.g., morning, afternoon, or evening), which would provide readers with more precise information. Finally, most studies only detailed the monitoring of the intervention group but not how the control group was monitored. These limitations should be addressed in future research by protocols including the time of exercise and more data on the protocol for the children and adolescents in the control group.

## 5 Conclusion

The purpose of this study was to synthesize the current literature on the relationship between sleep and physical activity in children and adolescents with neurodevelopmental disorders. Our results show that measuring sleep parameters by means of different measuring tools yields different results. The difficulties in interpreting many of the studies included in this meta-analysis were due to non-standardization of protocol, especially not including the ability range of the cohort, duration of the study, recommended exercises, whether the caregivers or researchers supervised the exercise regime/activity, and considering the practicality of continuing the exercise long-term by caregivers.

## Data Availability

The original contributions presented in the study are included in the article/supplementary material, further inquiries can be directed to the corresponding author.
